# Atypical glomus tumor arising in the liver: a case report

**DOI:** 10.1186/s13000-015-0355-4

**Published:** 2015-07-19

**Authors:** Katsutoshi Hirose, Takahiro Matsui, Hiroaki Nagano, Hidetoshi Eguchi, Shigeru Marubashi, Hiroshi Wada, Eiichi Morii

**Affiliations:** Department of Pathology, Osaka University Graduate School of Medicine, 2-2 Yamada-oka, Suita, Osaka 565-0871 Japan; Department of Gastroenterological Surgery, Osaka University Graduate School of Medicine, 2-2 Yamada-oka, Suita, Osaka 565-0871 Japan; Department of Digestive Surgery and Surgical Oncology, Yamaguchi University Graduate School of Medicine, 1-1-1 Minami-kogushi, Ube, Yamaguchi 755-8505 Japan; Department of Regenerative Surgery, Fukushima Medical University, School of Medicine, 1 Hikarigaoka, Fukushima, 960-1295 Japan

**Keywords:** Glomus tumor, Liver, Atypical features, Synaptophysin

## Abstract

**Background:**

Glomus tumors typically occur in the subcutaneous tissue of distal extremities, but rarely in visceral organs. Most glomus tumors are benign, while others have been reported to have malignant potential. Herein, a unique case of a liver glomus tumor with atypical histological features is reported.

**Case presentation:**

A 39-year-old man felt fullness in the epigastrium, and an enhanced computed tomography (CT) scan of the abdomen and pelvis revealed a 21-cm solid and cystic mass in the left liver lobe. The patient underwent a left hepatic lobectomy, and the tumor was pathologically identified as a glomus tumor with atypical histological features in the liver. This case is unique for three reasons. First, cases of glomus tumors in the liver are extremely rare. Second, this is the first report of a hepatic glomus tumor with histologically atypical features. Third, immunohistochemical staining showed focal positivity for synaptophysin. A literature review revealed that glomus tumors in visceral organs positive for synaptophysin show histological atypical features in most cases.

**Conclusions:**

This is the first case of a glomus tumor with atypical histological features arising in the liver. This unique case and literature review yielded interesting findings and enabled us to postulate that synaptophysin positivity may be indicative of atypical histological features in glomus tumors arising in visceral organs.

## Background

A glomus tumor is an uncommon mesenchymal perivascular tumor considered a vascular hamartomatous derivative of glomus bodies responsible for the thermoregulation of distal extremities. Most glomus tumors are diagnosed in patients in their fourth to sixth decades of life, although symptoms are often present for several years prior to diagnosis. Radiographic features of glomus tumors are not characteristic, and the supplementation of clinical impressions with radiographic studies is uncommon [1]. Glomus tumors typically occur in the skin of extremities but rarely in visceral organs. Glomus tumors in visceral organs are discovered incidentally or due to vague symptoms [1]. Most glomus tumors are benign, but some with malignant potential have been reported. Herein, we report a case of glomus tumor with atypical features arising in the liver.

## Case presentation

**Fig. 1:**
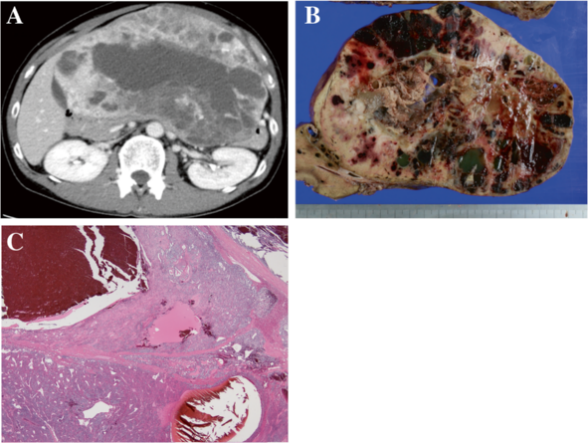
Enhanced CT, gross appearance, and tumor histology. Enhanced CT revealed a 21-cm solid and cystic mass in the left liver lobe (**a**). Cystic lesions filled with blood and solid lesions were observed (**b**). Very low-power field of the tumor histology (**c**, x5)

A 39-year-old Japanese male felt fullness in the epigastrium four months prior to admission. An enhanced computed tomography (CT) scan of the abdomen and pelvis revealed a 21-cm solid and cystic mass in the left liver lobe. The patient then visited the Department of Gastroenterological Surgery in our hospital for medical evaluation. Enhanced CT (Fig. 1a) revealed numerous tumor vessels in a bulky mass and cystic lesions filled with blood. Enhancement was observed in solid lesions and became stronger in the delayed phase. The left branch of the portal vein was closed due to compression or tumor invasion. The main portal and hepatic veins were also narrowed. No lesion was observed in the right lobe. Fluorodeoxyglucose-positron emission tomography (PET) revealed no other lesions or lymph node swelling. From these findings, a diagnosis of primitive undifferentiated sarcoma was considered, but a mesenchymal hamartoma or a sarcoma derived from hamartoma could not be excluded. According to serological tests, the levels of biliary tract enzymes were elevated, but those of hepatic transaminase and bilirubin were normal. After examination, the patient underwent left hepatic lobectomy.

## Pathological findings

**Fig. 2:**
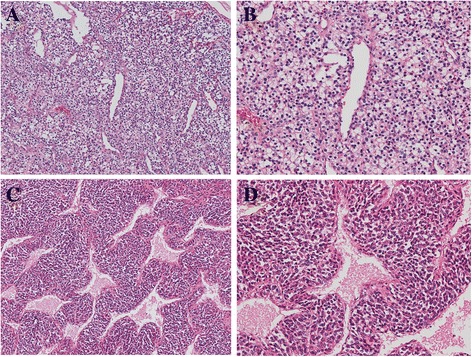
Histology of tumors in cystic (**a** and **b**) and solid (**c** and **d**) lesions. Monomorphic cells with round-to-oval nuclei and a pale cytoplasm and lacework of the basement membrane material were detected (**a**, x50; **b**, x150). In contrast, the tumor in the solid lesion was composed of cells with mild-to-moderate nuclear atypia and eosinophilic cytoplasm surrounded by vessels with a hemangiopericytomatous growth pattern (**c**, x50; **d**, x150)

**Fig. 3:**
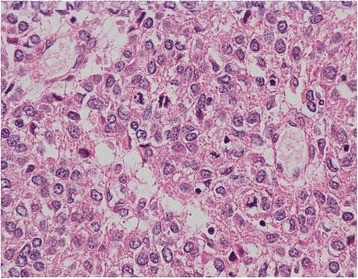
Histology of tumors in the solid lesion. High cellularity and frequent mitotic figures were observed (x400)

**Fig. 4:**
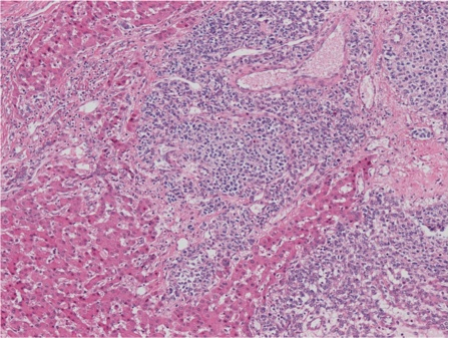
Histology of tumor margins showing an infiltrative growth pattern (x50)

Two components were macroscopically observed in the hepatic mass with a 20-cm diameter: a cystic lesion filled with blood and a white solid lesion (Fig. 1b). The background liver was normal. Low-power microscopic examination revealed that the tumor in the cystic lesions consisted of numerous blood vessels of varying size (Fig. 1c). The tumor was composed of monomorphic cells with round-to-oval nuclei and a pale cytoplasm, and basement membrane lacework material was detected around the cells (Fig. 2a). Cell membranes were well-defined, and few mitoses were observed (Fig. 2b). In contrast, the tumor in the solid lesions was composed of cells with mild-to-moderate nuclear atypia and the eosinophilic cytoplasm was surrounded by vessels with a hemangiopericytomatous growth pattern (Figs. 2c and d). High cellularity and frequent mitotic figures (35 mitotic figures per 50 high-power fields [HPFs]) were also observed (Fig. 3). Tumor margins showed an infiltrative growth pattern (Fig. 4), but no vascular invasion, perineural invasion, necrosis, or atypical mitosis was detected.

**Fig. 5:**
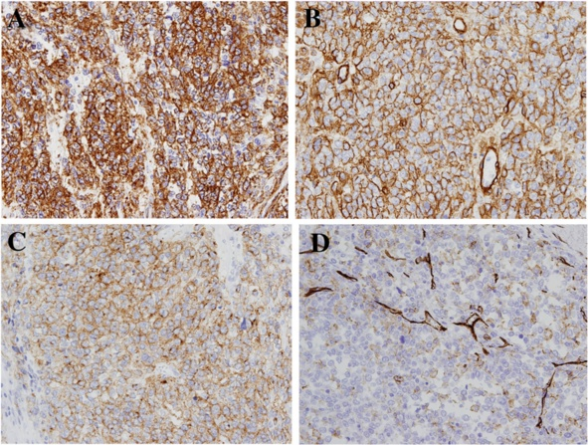
Immunohistochemical analysis of the tumor. Sections were stained with anti-SMA (clone 1A-4, 1:100, Dako, Carpinteria, CA), anti-collagen IV (clone CIV22, 1:50, Dako), anti-synaptophysin (clone 27G12, 1:200, Leica, Buffalo Grove, IL), and anti-CD31 (clone JC70A, 1:40, Dako) antibodies. Tumor cells were positive for SMA (**a**, x200), and collagen IV signal was distributed among tumor cells in a chicken-wire pattern (**b**, x200). Tumor cells were focally positive for synaptophysin (**c**, x200). CD31 staining showed faint positivity (**d**, x200)

Immunohistochemical (IHC) staining of the tumor was positive for both vimentin and smooth muscle actin (SMA) (Fig. 5a). The collagen IV signal was distributed among tumor cells in a chicken-wire pattern (Fig. 5b). Tumor cells were focally positive for calponin and synaptophysin (Fig. 5c). CD31 showed faint positivity (Fig. 5d), but CD34 staining was negative. Ets-related gene (ERG), Desmin, S100, low-molecular-weight cytokeratin (CAM5.2), pan-cytokeratin (AE1/AE3), cytokeratin 7, cytokeratin 19, CD117, chromogranin A, discovered on GIST (DOG-1), melanosome (HMB-45), CD56, alpha-fetoprotein (AFP), and hepatocyte paraffin 1 (HepPar-1) were completely negative. Both monomorphic cells in the cystic lesions and atypical cells in the solid lesions showed similar immunohistochemical results. The Ki-67 proliferative index was less than 3 % in cystic lesions and 15 % in solid lesions. From these findings, the tumor was diagnosed as a glomus tumor with atypical histological features.

## Discussion

Glomus tumors usually occur in the skin of extremities and are rarely present in visceral organs, such as the gastrointestinal tract [2], mediastinum [3], bladder [4], kidney [5], or corpus cavernosum [6]. Glomus tumors arising in the liver are extremely rare. To our knowledge, only six cases have been reported in the English literature, including the case presented herein [7–12].

The liver is the main target organ of metastasis. However, inspection of the present case prior to operation revealed no tumors in organs other than the liver, suggesting the tumor was not a metastatic lesion, but rather a primary tumor. It is necessary to distinguish glomus tumors from other tumor types, such as hemangioendotheliomas, gastrointestinal stromal tumors (GIST), paragangliomas, PEComas, and neuroendocrine tumors (NET). The lack of CD34 and ERG immunopositivity in conjunction with the histological appearance ruled out hemangioendothelioma. GIST and paraganglioma appeared to be ruled out because the tumor was CD117-, DOG-1-, S100-, and chromogranin A-negative. Concomitant positivity for synaptophysin and lack of melanosome positivity ruled out PEComa. NET is typically synaptophysin-, chromogranin A-, CD56-, and cytokeratin-positive. The present case was focally positive for synaptophysin, but negative for chromogranin A, CD56, CAM5.2, cytokeratin AE1/AE3, cytokeratin 7, and cytokeratin 19. The lesion was unlikely a hepatocellular carcinoma or hepatoblastoma, since both AFP and HepPar-1 were negative. Taken together, the findings suggest that the current tumor was a glomus tumor.

Glomus tumors are usually benign, but unusual histological features, including an infiltrative growth pattern, mitotic activity, and nuclear pleomorphism, have been reported in several cases. Folpe et al. analyzed 52 cases of glomus tumors with atypical histological features and defined a ‘malignant glomus tumor’ as that fulfilling at least one of the following criteria: (i) deep location and a size of more than 2 cm, (ii) atypical mitotic figures, or (iii) moderate-to-high nuclear grade and mitotic activity (more than 5 mitotic figures per 50 HPFs) [13]. They defined the criteria based on the fact that metastatic lesion was observed in 38 % of tumors fulfilling the criteria, while metastasis was not seen in tumors not classified as ‘malignant glomus tumors’ [13]. The problem with these criteria is that all glomus tumors arising in the liver meet the first criteria, since they are located in a deep space regardless of histological grade. Moreover, the glomus tumors reported in the liver to date are much larger than ordinary cutaneous glomus tumors (usually less than 1 cm). Kihara et al. stated that glomus tumors arising in visceral organs may be diagnosed belatedly because they have less intense symptoms and are less palpable [12]. Folpe et al. analyzed only three cases of visceral organ tumors (two in the lungs and one in the stomach), but did not analyze those without histological atypia [13]. Therefore, in agreement with Kihara et al. [12], we believe that liver glomus tumors cannot be classified as malignant on the basis of their location and size alone, and that histological evaluation described in the other two criteria (ii) and (iii) should be of value. None of the five hepatic glomus tumor cases reported thus far have displayed histological abnormalities [7–12]; this is the first case arising in the liver with histological atypical features. In the atypical-looking lesion, mitotic figures were frequently seen (35/50 HPFs), but nuclear atypia was not higher than a typical malignant glomus tumor [14]. Atypical mitotic figures were not evident in the present case. Taken together, while the present case showed an invasive growth pattern and high mitotic figures, the tumor cells did not entirely meet the second the third (ii and iii) histological criteria listed above, and we therefore concluded that the current tumor was not a malignant glomus tumor.

One of the most interesting features of the present case was that the tumor cells were focally positive for synaptophysin. In general, glomus tumors in peripheral soft tissues are synaptophysin-negative, but reports of glomus tumors focally positive for synaptophysin have been documented [5, 14]. Interestingly, glomus tumors positive for synaptophysin occur not in peripheral soft tissues, but in visceral organs [2]. More importantly, most of the reported glomus tumors positive for synaptophysin have atypical histological features. Song et al. reported a case of a malignant glomus tumor in the stomach that showed focal positivity for synaptophysin with prominent nuclear atypia and a fulminant course with multiorgan metastases [14]. Zhang et al. also reported a malignant glomus tumor of the esophagus with mediastinal lymph node metastases that tested positive for synaptophysin and exhibited increased high mitotic activity [15]. Moreover, other studies have reported cases of glomus tumors with atypical histological features and synaptophysin positivity in the kidney [5, 16], esophagus [17] and bronchus [18]. Although there is one report of a case of glomus tumor without atypia that stained positive for synaptophysin [19], most cases of glomus tumors positive for synaptophysin exhibit either histological atypia or clinically malignant behavior, such as metastasis. These previous reports and the present case suggest that synaptophysin positivity may correlate with atypical histological features in glomus tumors arising in visceral organs.

## Conclusions

In conclusion, we report an extremely rare case of a glomus tumor in the liver. Glomus tumors arising in visceral organs are very rare, as are those with atypical histological features, and little is known about the clinical course or prognosis of an uncommon glomus tumor. The types of glomus tumors in visceral organs with malignant courses, including metastasis, are also unknown. An accumulation of evidence regarding uncommon glomus tumors is indispensable to provide appropriate treatment and precisely estimate prognoses.

## Consent

Written informed consent was obtained from the patient for publication of this case report and any accompanying images. A copy of written consent is available for review with the Editor-in-Chief of this journal.
